# Quantitative Evaluation of Display Contrast of Gd-EOB-DTPA-Enhanced Magnetic Resonance Images: Effects of the Flip Angle and Grayscale Gamma Value

**DOI:** 10.1155/2019/7630671

**Published:** 2019-01-16

**Authors:** Yusuke Inoue, Hirofumi Hata, Ai Nakajima, Keiji Matsunaga, Yusuke Ohzeki, Tetsuya Hashimoto, Hiroki Haradome

**Affiliations:** ^1^Department of Diagnostic Radiology, Kitasato University School of Medicine, Sagamihara, Kanagawa, Japan; ^2^Department of Radiology, Kitasato University Hospital, Sagamihara, Kanagawa, Japan; ^3^Department of Diagnostic Radiology, Kitasato University Hospital, Sagamihara, Kanagawa, Japan

## Abstract

**Introduction:**

Display contrast can be changed nonlinearly by manipulating the gamma value of the grayscale. We investigated the contrast of the hepatobiliary-phase images acquired with different flip angles (FAs) and displayed with different gamma values in Gd-EOB-DTPA-enhanced magnetic resonance imaging.

**Material and Methods:**

Twenty patients with liver tumors were studied. Hepatobiliary-phase images were acquired at low (12°) and high (30°) FAs. Low-FA images were converted to simulate images displayed with different gamma values, using ImageJ software. To assess image contrast, the liver-to-muscle signal ratio (LMR), liver-to-spleen signal ratio (LSR), contrast ratio (CR), liver signal-to-noise ratio (SNR), and contrast-to-noise ratio (CNR) were calculated.

**Results:**

The LMR, LSR, and CR were higher in the high-FA images than in the low-FA original images. Although the SNR was lower in the high-FA images, indicating an increase in noise, the CNR was higher. Raising the gamma value increased the LMR, LSR, and CR, notably decreased the SNR, and slightly decreased the CNR.

**Conclusion:**

Increasing the FA enhanced image contrast, supporting its usefulness for improving the delineation of focal liver lesions. Although the associated increase in noise may be problematic, raising the grayscale gamma value enhances the display contrast of low-FA images.

## 1. Introduction

Gadolinium ethoxybenzyl-diethylenetriamine pentaacetic acid (Gd-EOB-DTPA) is a hepatobiliary contrast agent for magnetic resonance (MR) imaging. The agent is selectively taken up by hepatocytes and excreted via the biliary system. Typically, in addition to acquisition of dynamic images at the time of intravenous administration, hepatobiliary-phase imaging is performed 20 min later. Gd-EOB-DTPA enhances the signal of liver parenchyma on the hepatobiliary-phase images and increase the contrast between liver parenchyma and focal liver lesions, facilitating lesion detection [1, 2].

Gd-EOB-DTPA-enhanced MR imaging usually utilizes a T1-weighted gradient echo sequence, in which the flip angle (FA), together with the repetition time, is a key parameter to determine image contrast. Increasing the FA has been demonstrated to increase the contrast of the hepatobiliary-phase images and to improve the detectability and conspicuity of focal liver lesions [3, 4]. Acquisition of high-FA hepatobiliary-phase images is accepted to be a useful adjunct to the acquisition of dynamic and hepatobiliary-phase images using a low FA.

Medical images, including MR images, are displayed on the monitors of picture archiving and communication system (PACS) viewers for visual image interpretation. The interpreter can adjust the display conditions, which are crucial for visual interpretation and thus for clinical diagnosis. Display conditions are usually manipulated using the window width and window level. The window width is the difference between the upper and lower limits of the display window; display contrast can be enhanced by narrowing it, with no need for additional data acquisition. In computed tomography, the pixel values expressed in Hounsfield units are calibrated and the optimal window setting can be standardized. In contrast, the signal intensities of MR images vary greatly depending on scanners, imaging parameters, and patients [5]. Although standardization of window settings has been attempted [6–8], interpreters commonly adjust the display conditions during interpretation manually and subjectively, which increases interpreter's burden and dependency on interpreter's skill.

Manipulating the gamma value of the grayscale also changes image appearance on the monitors. The gamma value determines the grayscale pattern, and the relationship between the pixel value and brightness on the monitor can be changed nonlinearly by manipulating the gamma value. A gamma value of 1 corresponds to an ordinary linear scale. Raising the gamma value increases the contrast between regions with high and medium pixel values, while decreasing the contrast between regions with medium and low pixels values, and would be expected to enhance the contrast between liver parenchyma and focal liver lesions. Although the grayscale pattern of the display device itself is usually fixed to the Grayscale Standard Display Function (GSDF), defined in Digital Imaging and Communications in. Medicine (DICOM) part 14, in the PACS viewer, software-based manipulation of each image set is possible using software of the viewer.

We routinely acquire low-FA images during the dynamic phase and both low-FA and high-FA images during the hepatobiliary phase in Gd-EOB-DTPA-enhanced MR imaging. In the present preliminary study, we quantitatively evaluated the contrast of the hepatobiliary-phase images acquired at low and high FAs. The contrast of low-FA images was assessed in relation to the grayscale gamma value. The aim of this study was to examine the potential of high-gamma display as a method of improving image contrast in Gd-EOB-DTPA-enhanced MR imaging.

## 2. Material and Methods

### 2.1. Patients

Data from 20 patients (17 men and 3 women; 68.0 ± 11.1 years, mean ± SD) who underwent Gd-EOB-DTPA-enhanced MR imaging using a 1.5T scanner for clinical indications from January 2015 to July 2015 were analyzed retrospectively. Patients in whom untreated liver tumors (hepatocellular carcinoma 13, metastatic liver tumor 7) of 2 cm or larger were identified on hepatobiliary-phase images were included. The exclusion criteria were (1) poor breath holding, (2) prior liver resection, (3) extensive liver tumors, (4) prior splenectomy, and (5) severe atrophy of the erector muscles of spine, and otherwise, patients were enrolled consecutively. The criteria (2)-(5) were defined to allow appropriate setting of regions of interest (ROIs). Of the 20 patients, ten had liver cirrhosis, one had chronic hepatitis, one had primary biliary cirrhosis, and eight normal background livers. The study protocol was approved by the Institutional Review Board, and the need for informed consent was waived.

### 2.2. Imaging Procedures

MR imaging was performed using a 1.5T clinical scanner (Signa HDxt; GE Healthcare, Waukesha, WI) with a 12-channel phased-array coil. With intravenous administration of Gd-EOB-DTPA (0.025 mmol/kg; Bayer Yakuhin, Osaka, Japan), dynamic and hepatobiliary-phase images were acquired in the axial plane using liver acquisition with volume acceleration (LAVA) sequence, during a single breath hold at expiration.

Hepatobiliary-phase images were obtained at an FA of 12° (low FA) and then at an FA of 30° (high FA). Other imaging parameters were typically as follows: repetition time = 4.3 ms (low FA) or 5.6 ms (high FA), echo time = 2.0 ms (low FA) or 2.2 ms (high FA), field of view = 360 mm, matrix = 320 × 192, slice thickness = 5 mm, slice number = 44, and acquisition time = 16 s (low FA) or 21 s (high FA). Repetition time and echo time differed between low-FA and high-FA imagings because they were determined automatically depending on the FA. True spatial resolution was 1.1 × 1.9 × 5.0 mm^3^, and reconstructed spatial resolution was 0.7 × 0.7 × 2.5 mm^3^. Field of view and slice number were increased as required in large patients. A parallel imaging technique (array spatial sensitivity encoding technique [ASSET]) was used with a reduction factor of 2. Image uniformity correction was performed using phased-array uniformity enhancement (PURE).

### 2.3. Image Analysis

Images were analyzed using ImageJ software ver. 1.48v (National Institutes of Health, Bethesda, MD). The pixel values of low-FA images were converted using the gamma correction function of the software, and stored. The gamma values were set at 1.2, 1.5, and 1.8 to produce gamma-1.2, gamma-1.5, and gamma-1.8 images, respectively. The original images corresponds to images with a gamma value of 1.0. Although image display can be changed on a PACS monitor by manipulating the gamma value of the grayscale, the pixel values are not affected by such manipulation. We created and stored images after gamma correction, simulating images displayed with different gamma values, to quantitatively assess image contrast.

Signal intensities were measured in the low-FA images with different gamma values and in the high-FA original images. ROIs were placed in the liver parenchyma, spleen, muscle, and liver tumors. For the liver parenchyma, a circular ROI of 100 mm^2^ was placed in each of the anterior segment of the right hepatic lobe, the posterior segment of the right lobe, and the medial segment of the left lobe, avoiding vessels, focal liver lesions, and imaging artifacts, on the slice exhibiting the right main branch of the portal vein. The means and SDs of the signal intensities in the ROIs were averaged among the three ROIs to obtain liver signal and liver noise, respectively. For the muscle, an elliptical ROI of 100 mm^2^ was placed in each of the right and left paravertebral muscles, minimizing inclusion of fat, on the slice used to assess liver signals. Muscle signal was defined as the average of the mean signal intensities in the right and left ROIs. A circular ROI of 200 mm^2^ was placed in the spleen, and spleen signal was defined as the mean signal intensity in the ROI. As for the liver tumor, the largest tumor was selected for analysis when two or more tumors were present in a given patient, and an ROI of 100 mm^2^ was placed on a slice exhibiting the largest cross-section of the tumor. The dominant component showing homogenous intensity was assessed when the signal intensity of the tumor was heterogeneous. Tumor signal was defined as the mean signal intensity in the ROI.

To assess image contrast, the liver-to-muscle signal ratio (LMR), liver-to-spleen signal ratio (LSR), contrast ratio (CR), liver signal-to-noise ratio (SNR), and contrast-to-noise ratio (CNR) were calculated for each image set as follows:

  LMR = liver signal/muscle signal
  LSR = liver signal/spleen signal
  CR = (liver signal − tumor signal)/liver signal
  SNR = liver signal/liver noise
  CNR = (liver signal − tumor signal)/liver noise.

### 2.4. Statistical Analysis

Values are expressed as means ± SDs. Indices of image contrast were compared statistically among low-FA original images, low-FA gamma-1.8 images, and high-FA original images. For parametric distribution, comparison was made by one-way repeated analysis of variance, and post hoc analysis was performed by the paired t test with Holm*ʼ*s correction. For nonparametric distribution, the Friedman*ʼ*s test was used, followed by the Wilcoxon signed rank test with Holm*ʼ*s correction for post hoc analysis. A p value of less than 0.05 was deemed to be statistically significant.

## 3. Results

**Figure 1:**
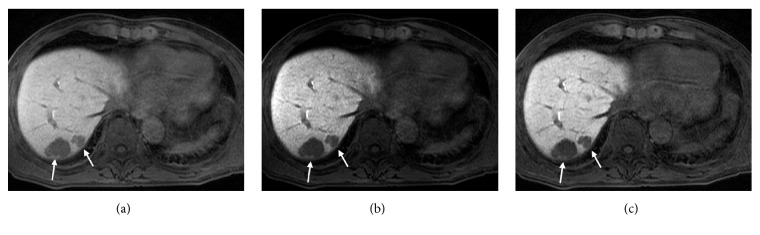
A low-FA original image (a), a low-FA gamma-1.8 image (b), and a high-FA image (c) in a 66-year-old man with metastatic liver tumor (arrows).

**Figure 2:**
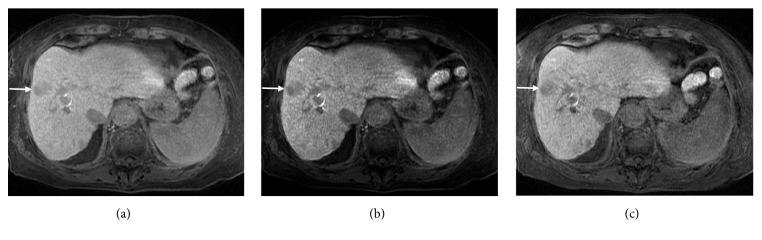
A low-FA original image (a), a low-FA gamma-1.8 image (b), and a high-FA image (c) in a 73-year-old woman with hepatocellular carcinoma (arrows) and liver cirrhosis.

**Figure 3:**
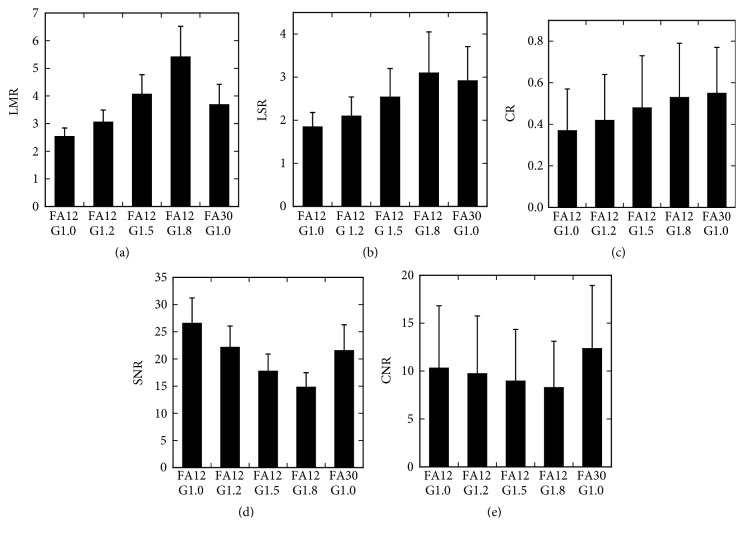
Results of quantitative indices: LMR (a), LSR (b), CR (c), SNR (d), and CNR (e). Error bars indicate SDs. FA12 and FA30 represent FAs of 12° and 30°, respectively. G1.0, G1.2, G1.5, and G1.8 represent the gamma values of 1.0, 1.2, 1.5, and 1.8, respectively.

The high-FA images provided higher image contrast visually between the liver and muscle, the liver and spleen, and liver parenchyma and liver tumor, when compared with the low-FA original images (Figures 1 and 2). On quantitative analysis, the LMR, LSR, and CR were higher in the high-FA images than in the low-FA original images (Figure 3). Although the SNR was lower in the high-FA images, indicating an increase in noise, the CNR was higher.

Raising the gamma value of the grayscale enhanced image contrast visually. The LMR, LSR, and CR increased with the gamma value. As the gamma value increased, the SNR decreased notably, indicating an increase in noise, and the CNR decreased slightly.

**Figure 4:**
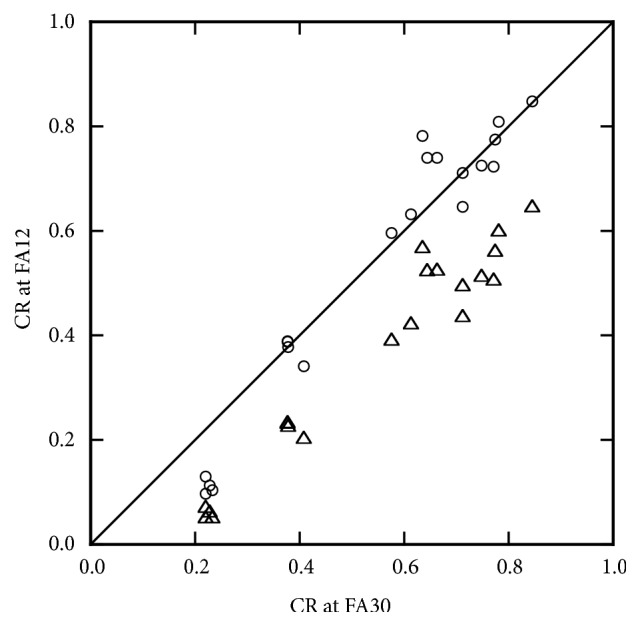
Relationship between CRs determined at FAs of 30° (FA30) and 12° (FA12). CRs obtained at an FA of 12° are presented for gamma values of 1.0 (open triangles) and 1.8 (open circles), respectively. The solid line represents the line of identity.

The CR in the low-FA gamma-1.8 images was comparable to that in the high-FA images, and this value was selected for further analysis. The low-FA original images, and low-FA gamma-1.8 images, and high-FA images were subjected to statistical evaluation. The differences in all five indices between the high-FA images and low-FA original images were statistically significant. The CR did not differ significantly between the high-FA images and low-FA gamma-1.8 images, and was lower in the low-FA original images (Figure 4). The LMR and LSR was greatly and slightly higher in the low-FA gamma-1.8 images than in the high-FA images, respectively, with statistical significance. The SNR and CNR were significantly lower in the low-FA gamma-1.8 images.

## 4. Discussion

We quantitatively evaluated the image contrast of hepatobiliary-phase images in Gd-EOB-DTPA-enhanced MR imaging, with special reference to the potential usefulness of manipulating the gamma value of the grayscale for monitor display. Increasing the FA in image acquisition has been demonstrated to increase image contrast and to improve the delineation of focal liver lesions [3, 4]. In the present study, increasing the FA from 12° to 30° increased image contrast, as indicated by higher LMR, LSR and CR values, in line with the previous studies. Increasing the FA reduced the CR because of an increase in noise; however, the increase in contrast overrode the noise increase, resulting in a higher CNR. These results, increase in both the CR and CNR, supports usefulness of high-FA imaging to improve the delineation of focal liver lesions in hepatobiliary-phase images.

Imaging at a different FA provides a different image set. Even a given image set has a different appearance on the monitor when a different grayscale is used. The gamma value of the grayscale defines the relationship between the pixel value and brightness on the monitor, which can be changed nonlinearly by manipulating the gamma value. In the present study, raising the gamma value increased the LMR, LSR, and CR, indicating an increase in contrast. Application of a gamma value of 1.8, instead of 1.0, to low-FA images yielded LSR and CR values comparable to, and an LMR value higher than, those in the high-FA images. On the other hand, because raising the gamma value increases differences in brightness between high-value and medium-value pixels nonspecifically, this manipulation caused evident noise increase, as indicated by the reduction in the SNR. Unlike increasing the FA, raising the gamma value decreased the CNR. The CNR was lower in low-FA gamma-1.8 images than in high-FA images. Display of low-FA images with the gamma value of 1.8 is indicated to provide image contrast comparable to that of high-FA images, with no need for additional data acquisition; however, poorer noise property may hamper image interpretation. The usefulness of this display method may depend on various factors such as the MR scanner, imaging sequence, status of background liver, and nature of focal liver lesions. The quantitative analysis of image characteristics in this preliminary study employing a small number of patients indicates that the method may be useful but the usefulness is not conclusive, and further studies using visual interpretation of many clinical images are warranted.

In this study, we created and stored additional images using the gamma correction function of ImageJ software to assess image characteristics quantitatively. Such processing is not required when high-gamma display is used for visual interpretation. Enhancement of the display contrast of low-FA images is easily achieved on a PACS viewer by manipulating the gamma value; it is unnecessary to process the original data. This is similar to adjustment of display conditions using the window width and window level. Adjustment using the width and level requires trial and error by the image interpreter to find an optimal combination of these two parameters and may be troublesome especially for inexperienced interpreters [9]. Since the signal intensities are affected by various factors, an optimal combination of the width and level cannot be fixed. Application of a fixed gamma value for enhancement of display contrast may contribute to improving efficiency and reproducibility in optimizing image display, which would have a substantial impact on clinical radiology practice. High-FA imaging offers better contrast than low-FA imaging and the hepatobiliary-phase imaging is often performed at both high and low FAs. However, repetitive imaging at different FAs is not applicable to dynamic imaging because of rapid changes in Gd-EOB-DTPA distribution early after injection. Only conventional low-FA images are usually acquired during the dynamic phase. Manipulation of the gamma value does not require an additional data acquisition and is expected to have a potential of improving assessment of vascularity on dynamic images.

## 5. Conclusions

We investigated methods for improving the contrast of hepatobiliary-phase images in Gd-EOB-DTPA-enhanced MR imaging. Increasing the FA increased image contrast, supporting its usefulness in improving the delineation of focal liver lesions. Although the increase in noise may be problematic, raising the gamma value of the grayscale is indicated to improve the display contrast of low-FA images conveniently. Manipulation of display contrast using the grayscale gamma value may aid in visual evaluation of focal liver lesion and is worth further evaluation.

## Data Availability

The data used to support the findings of this study are available from the corresponding author upon request.
